# Extracellular Vesicle-Based Biomarkers in Spinal Cord Injury: A State-of-the-Art Review on Diagnostic and Prognostic Advances

**DOI:** 10.3390/ijms27042079

**Published:** 2026-02-23

**Authors:** Trung Nhan Vo, Hae Eun Shin, Yeji Kim, Inbo Han

**Affiliations:** 1 Department of Biomedical Science, CHA University, Seongnam-si 13488, Republic of Korea; 2 Department of Neurosurgery, CHA Bundang Medical Center, CHA University, Seongnam-si 13496, Republic of Korea; 3 School of Medicine, CHA University, Seongnam-si 13496, Republic of Korea

**Keywords:** spinal cord injury, extracellular vesicles, liquid biopsy, biomarkers, microRNA, diagnosis, prognosis

## Abstract

Spinal cord injury (SCI) is a devastating neurological disorder that can result in permanent disability and reduced quality of life, characterized by heterogeneous injury mechanisms and limited tools for accurate early diagnosis and prognostic stratification. The clinical course of SCI is driven not only by the initial mechanical insult but also by complex secondary injury cascades involving neuroinflammation, axonal degeneration, demyelination, and maladaptive repair responses. Current diagnostic and prognostic approaches, which rely largely on neurological examination and imaging, provide limited insight into these dynamic molecular processes. In this context, extracellular vesicles (EVs) have emerged as a biologically compelling source of biomarkers for SCI. EVs are released by neurons, glial cells, endothelial cells, and immune cells and carry molecular cargo that reflects cellular stress, injury severity, and endogenous repair activity. Increasing evidence indicates that EV-associated proteins and regulatory microRNAs (miRNAs) encode injury-specific signatures related to neuronal and glial damage, inflammatory signaling, metabolic stress, and functional recovery potential. In this review, we summarize the current knowledge on EV biology in SCI and discuss emerging evidence supporting EV-derived proteins and miRNAs as promising tools for refining diagnosis and prognosis. Our aim is not only to consolidate established findings but also to highlight EV-based molecular signatures as a developing framework for precision biomarker discovery in SCI.

## 1. Introduction

Spinal cord injury (SCI) is a neurological disorder that is most commonly caused by acute traumatic injury but can also result from non-traumatic pathologies. This life-threatening condition is typically characterized by abrupt and severe loss of motor and sensory function and autonomic nervous system function below the level of the lesion [1,2]. The pathophysiology of SCI is initiated by mechanical impact, which is defined as the primary injury. Immediately thereafter, secondary injury occurs as a cascade of time-dependent molecular and cellular reactions [3,4], including ischemia, neuroinflammation, oxidative stress, and excitotoxicity, which collectively expand the area of functional impairment [5]. Through this process, SCI causes severe and lifelong functional disability. SCI is also a major public health issue that contributes to a substantial socioeconomic burden [6].

Detailed diagnosis and early prognosis are important for successful acute-phase management and for planning long-term rehabilitation. Current clinical assessment mainly relies on the International Standards for Neurological Classification of Spinal Cord Injury and the American Spinal Injury Association (ASIA) Impairment Scale. Radiological examinations, such as magnetic resonance imaging (MRI), are also employed for clinical assessment [7]. Across patients with SCI, including those with incomplete SCI, substantial heterogeneity exists, and variability in neurological recovery is observed. Most clinically meaningful motor function recovery occurs early and typically reaches a plateau within 12 months post-SCI [8]. A key challenge in current clinical assessment is precisely predicting the extent of neurological functional recovery during the acute phase. Accurate prediction enables patient stratification for clinical trials and the development of personalized treatment regimens [9]. Therefore, there is an unmet need to discover novel, reliable biomarkers that can noninvasively track secondary injury processes and accurately predict long-term neurological functional prognosis.

Extracellular vesicles (EVs) have recently been highlighted as promising candidates to address current limitations in diagnosis and prognosis prediction for SCI and to meet unmet needs [10]. EVs are heterogeneous, cell-derived, membrane-bound nanoparticles. They act as key mediators of intercellular communication [11] by transferring diverse molecular cargo, including proteins, lipids, and nucleic acids—particularly microRNAs (miRNAs)—that can accurately reflect the physiological and pathological state of their parent cells [12]. The potential of EVs as biomarkers for SCI diagnosis and prognosis is supported by two key advantages. First, EVs are abundant and stable in accessible biological fluids such as plasma, serum, and cerebrospinal fluid (CSF) [13], making them suitable tools for liquid biopsy-based clinical management. Second, EVs secreted from central nervous system (CNS) cells can cross the blood–brain barrier and potentially the blood–spinal cord barrier [14]. Thus, EVs can deliver a molecular snapshot of pathophysiological states within the injured spinal cord to the peripheral circulation [15]. This characteristic provides a noninvasive window for monitoring disease progression and molecular mechanisms linked to recovery.

**Figure 1:**
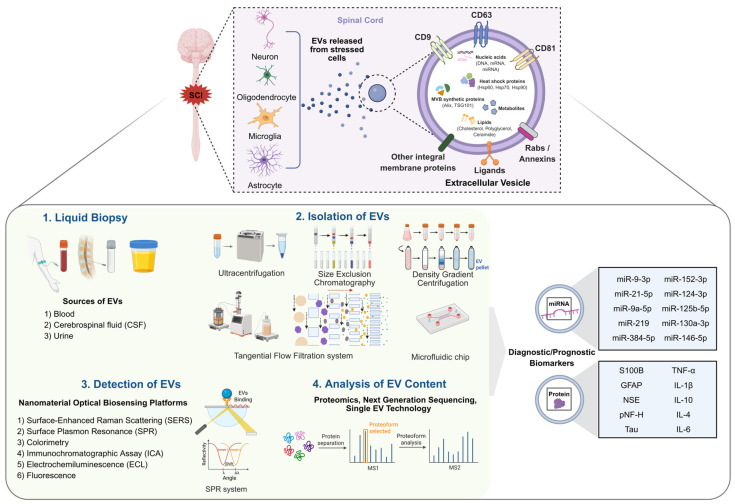
Extracellular vesicle-based biomarker workflow in spinal cord injury (SCI). This figure provides a conceptual framework for identifying EV-derived biomarkers in SCI. Following SCI, multiple cell types within the injured microenvironment, including macrophages, microglial cells, astrocytes, oligodendrocytes, and neural stem cells, release EVs containing molecular cargo such as mRNA, DNA, proteins, and miRNAs. These EVs can be obtained through liquid biopsy from various bodily fluids, including blood, urine, and cerebrospinal fluid. After sample collection, EVs are isolated using established approaches such as ultracentrifugation, size-exclusion chromatography, density-gradient separation, tangential flow filtration, or emerging microfluidic platforms. Downstream detection and molecular profiling can be achieved using conventional and advanced technologies, including high-resolution multiomics strategies such as next-generation sequencing and proteomics. The identified EV-derived miRNAs and proteins may have diagnostic and prognostic value, offering promising biomarkers for evaluating injury severity, monitoring disease progression, and guiding personalized therapeutic strategies in SCI. Abbreviations: EV, extracellular vesicle; SCI, spinal cord injury; CSF, cerebrospinal fluid; miRNA, microRNA; mRNA, messenger RNA.

The potential of EV-based biomarkers in SCI is supported by evidence accumulated in other major CNS diseases [16]. In traumatic brain injury (TBI), changes in circulating exosomes (a subtype of EVs)—particularly brain-derived proteins and miRNA profiles—have been shown to correlate with injury severity and neurological outcomes [17]. Similarly, in neurodegenerative diseases such as Alzheimer’s disease and amyotrophic lateral sclerosis, specific molecular markers within EVs have been suggested as valuable biomarkers for early diagnosis and monitoring of disease progression [18,19]. This broader utility of EVs as noninvasive biological messengers across multiple neurotraumatic and neurodegenerative diseases supports the hypothesis that EVs and their molecular cargo represent a feasible avenue for discovering sensitive and clinically viable biomarkers for SCI diagnosis and prognosis (Figure 1) [20].

## 2. Extracellular Vesicles: Types, Characteristics, and Cargo

### 2.1. Types and Production of EVs

EVs represent a heterogeneous population of lipid bilayer-delimited particles released by virtually all cell types, ranging from prokaryotes to eukaryotes, into the extracellular space [21,22]. Historically perceived as repositories for cellular waste or by-products of homeostasis, EVs have been redefined over the last decade as pivotal mediators of intercellular communication by facilitating horizontal transfer of bioactive cargo, including proteins, lipids, and nucleic acids. EVs are capable of reprogramming the phenotype and function of recipient cells in both physiological and pathological contexts [22,23,24]. To fully appreciate their potential as biomarkers, it is first necessary to delineate the mechanisms governing their biogenesis, clarify nomenclature, and describe the molecular diversity of their cargo.

#### 2.1.1. Nomenclature and Classification Standards

EV classification has historically relied on biogenesis pathways, dividing EVs into three primary subtypes. First, exosomes were defined as vesicles of endocytic origin (30–150 nm) released via fusion of multivesicular bodies with the plasma membrane. Second, microvesicles (MVs; also termed ectosomes or microparticles) were described as forming through direct outward budding and fission of the plasma membrane, typically ranging from 100 nm to 1 μm, although they can be as small as 30 nm. Third, apoptotic bodies are large vesicles (1–5 μm) generated via membrane blebbing and fragmentation during programmed cell death. However, due to substantial overlap in physicochemical properties such as size, density, and surface marker expression, distinguishing these subpopulations in complex biofluids remains a formidable challenge. Consequently, the International Society for Extracellular Vesicles (ISEV), through the MISEV2023 guidelines, advises against the use of biogenesis-specific terms such as “exosome” unless subcellular origin can be experimentally verified [21,23,25].

Instead, the field is moving toward operational terminology based on physical characteristics. For example, particles can be classified by size into small EVs (<200 nm) and large EVs (>200 nm). In the context of biomarker discovery for SCI, it is critical to distinguish EVs from non-vesicular extracellular particles (NVEPs). The umbrella term extracellular particle encompasses both vesicular entities and non-vesicular assemblies lacking a lipid bilayer. Because NVEPs frequently co-isolate with EVs and may carry distinct molecular cargo, precise nomenclature is essential to attribute a diagnostic signal to vesicular versus non-vesicular components. For the purpose of this review, we use biogenesis-specific terms (e.g., exosome, microvesicle) when discussing mechanisms established in the literature and the generic term EVs when referring to heterogeneous populations typically recovered from biofluids [25].

#### 2.1.2. Biogenesis and Release Mechanisms

The diversity of EVs primarily arises from their distinct intracellular origins. The two dominant pathways of EV generation involve the endosomal network (exosomes) and plasma membrane budding (microvesicles).

Exosomes (30–150 nm) originate from the endocytic pathway. The limiting membrane of early endosomes undergoes inward budding, which sequesters cytosolic components and transmembrane proteins into intraluminal vesicles (ILVs). This process converts endosomes into multivesicular bodies (MVBs). Cargo sorting into ILVs is a tightly regulated process driven primarily by the Endosomal Sorting Complex Required for Transport (ESCRT) machinery; however, exosome formation can also proceed via ESCRT-independent mechanisms. Following maturation, MVBs are transported to the plasma membrane via the cytoskeletal network and microtubule motors [21,23,24].

In contrast, microvesicles (MVs; 100–1000 nm) are generated through direct outward budding and fission of the plasma membrane. This process requires substantial remodeling of the actin cytoskeleton and is mediated by molecular motors (e.g., myosins and kinesins) and small guanosine triphosphatases such as ADP-ribosylation factor 6 and RhoA. MV biogenesis also involves phospholipid translocation. Specifically, externalization of phosphatidylserine alters membrane curvature and rigidity, facilitating vesicle scission [21,23,24].

Apoptotic bodies (>1000 nm) are released exclusively by cells undergoing programmed cell death. As the cytoskeleton dissociates from the plasma membrane because of increased hydrostatic pressure, the membrane blebs and fragments. Unlike exosomes and MVs, apoptotic bodies frequently contain intact organelles, large chromatin fragments, and high concentrations of histones [21,22].

### 2.2. Profiling Content in EVs

EVs derive their biological activity and clinical significance from the complex and selective composition of their molecular cargo. This cargo profile contains information about the physiological and pathological state of the donor cell, making it a key target for developing biomarkers that reflect disease states such as SCI [26].

The internal contents of EVs, particularly exosomes, are not simple cytoplasmic effluents but are loaded into the lumen of MVBs through active, highly regulated cargo-sorting mechanisms. This sorting process directly influences EV composition. The contents are broadly categorized into four main groups: nucleic acids, proteins, lipids, and metabolites [26,27].

Protein cargo provides crucial information regarding EV origin and sorting pathways. In addition, the exosomal surface is enriched with characteristic tetraspanin family proteins such as CD9, CD63, and CD81, which serve as widely used surface markers for EV characterization. These proteins localize to lipid raft domains and participate not only in membrane organization and cargo sorting but also in recognition and binding to recipient cells [26,27].

EVs transport diverse nucleic acids, including DNA, mRNA, and noncoding RNAs. Notably, miRNAs are considered among the most functionally important EV cargo species. miRNAs are actively sorted and loaded through interactions with Argonaute 2 and specific RNA-binding protein complexes. Once delivered to recipient cells, EV-associated miRNAs alter gene expression by regulating translation of target mRNAs. In neurotrauma contexts such as SCI, these miRNAs function as key mediators of intercellular communication by activating or suppressing pathways related to inflammation, neuroprotection, and axonal regeneration.

Lipid and metabolite composition also influences cargo selection and EV stability. The exosomal membrane is enriched in cholesterol and sphingolipids (particularly ceramides) compared with the typical cell membrane, conferring increased structural stability. Ceramides, in particular, promote exosome formation by inducing membrane curvature in lipid-mediated ESCRT-independent pathways. This lipid composition also supports efficient fusion of EVs with recipient cell membranes. In addition, EVs contain diverse metabolites that reflect the metabolic state of the parent cell, providing potentially valuable information for tracking changes in local metabolic dysregulation or stress responses after neural injury [26,27,28].

Precise profiling of EV contents enhances understanding of SCI pathophysiology and can expand potential clinical applications. Analysis of miRNA expression patterns, injury-related proteins, and metabolic changes within EVs isolated from patient biofluids (e.g., blood and CSF) may provide noninvasive, sensitive biomarkers for assessing injury severity and recovery potential. In this review, we focus primarily on EV cargo components with demonstrated diagnostic or prognostic relevance in SCI cohorts, particularly miRNAs and structural proteins.

### 2.3. Advances in Extracellular Vesicle Isolation and Detection Technologies

#### 2.3.1. Isolation Techniques: From Classical to Innovative

The heterogeneity of EVs requires isolation strategies that balance recovery yield with purity. In the context of SCI, where sample volumes—particularly CSF—are often limited and the risk of protein contamination is high, selection of isolation methodology is critical for downstream diagnostic accuracy.

Traditional separation techniques have long supported early EV research but often present technical limitations for clinical implementation. Ultracentrifugation (UC), the historical benchmark, typically isolates EVs based on differences in size and density. However, UC carries a risk of co-precipitating non-vesicular contaminants. Moreover, high shear forces generated during high-speed centrifugation can compromise vesicle integrity and induce artificial aggregation, potentially distorting analysis of fragile neural biomarkers. Although density-gradient centrifugation improves purity, it markedly reduces recovery (<30%) and increases processing time, limiting feasibility for rapid clinical decision-making in acute trauma settings. In addition, high centrifugal forces may disrupt EV integrity. Size-exclusion chromatography (SEC) provides higher purity by isolating EVs based on size through porous columns and generally preserves EV structural integrity. Precipitation-based methods, such as polymer-mediated aggregation using polyethylene glycol to reduce solubility and deposit EVs, offer high throughput and operational simplicity suitable for low-volume clinical samples such as CSF. However, EV purity is often substantially lower because of co-precipitation of contaminating proteins [29].

Recent technological advances have emphasized scalability and precision, moving beyond bulk isolation toward higher-resolution subpopulation enrichment. Tangential flow filtration (TFF) represents a scalable evolution of conventional filtration. Unlike dead-end filtration, in which fluid is forced perpendicularly through a membrane and rapid clogging occurs, TFF directs fluid flow parallel to the filter surface. This cross-flow mechanism reduces membrane fouling and enables processing of larger sample volumes with reduced shear stress. TFF discriminates EVs based on size thresholds (molecular weight cutoffs), and when coupled with SEC (TFF–SEC), it provides a robust platform for producing EV preparations with improved purity and yield. This approach may be particularly useful for removing high loads of cellular debris present in acute SCI samples [29].

Microfluidic platforms are transforming EV isolation by integrating multimodal separation principles—such as size, affinity, acoustics, and viscoelasticity—within micrometer-scale channels. These systems offer several advantages for clinical diagnostics, including high sensitivity, rapid processing, and the ability to handle minute sample volumes. Innovations such as the EXODUS system, which uses harmonic oscillation and negative pressure, can achieve automated, label-free purification with purity reported to exceed that of UC. Similarly, acoustic trapping technologies and viscoelastic microfluidics enable sorting of EV subpopulations from complex matrices such as whole blood without labeling. By enabling multiparametric sorting, microfluidics facilitates dissection of EV heterogeneity, a critical step for identifying specific biomarkers in complex pathologies such as SCI [29,30].

#### 2.3.2. Nanomaterial-Enabled Optical Biosensing Platforms for Extracellular Vesicle Detection

**Figure 2:**
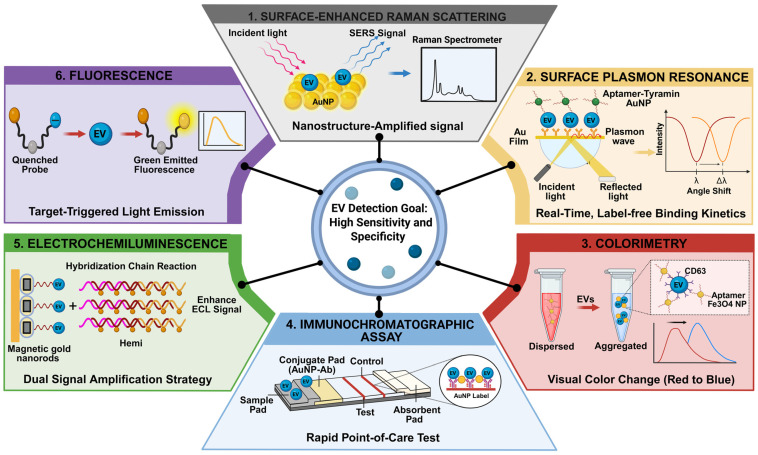
Nanomaterial-enabled biosensing platforms for high sensitivity and specificity in extracellular vesicle detection. Schematic overview of advanced optical and electrochemical technologies developed to achieve highly sensitive and specific detection of extracellular vesicles (EVs) in complex biofluids. Surface-enhanced Raman scattering (SERS) exploits plasmonic nanostructures to amplify EV-associated molecular fingerprints, enabling ultrasensitive spectral detection. Surface plasmon resonance (SPR) provides real-time, label-free monitoring of EV–ligand interactions through refractive index changes at metal surfaces. Colorimetric assays translate EV recognition into visible color shifts via nanoparticle aggregation, facilitating rapid, instrument-free detection. Immunochromatographic assays (ICA) enable point-of-care EV testing using nanoparticle-labeled antibodies for visual readout. Electrochemiluminescence (ECL) platforms combine nanomaterial-based signal amplification strategies to achieve low background and wide dynamic range. Fluorescence-based approaches use target-triggered or plasmon-enhanced emission to improve signal-to-noise ratios. Collectively, these complementary platforms address the central challenge of EV diagnostics by maximizing detection sensitivity and specificity for clinical biomarker applications.

Detection of EVs in SCI diagnostics requires sensors that are not only sensitive but also capable of discerning vesicle-specific signals within complex biological matrices. Nanomaterials can serve as powerful transducers that amplify signals and reduce background interference by leveraging properties such as localized surface plasmon resonance and catalytic activity (Figure 2).

Surface-enhanced Raman scattering (SERS): SERS amplifies vibrational signatures of analytes adsorbed on plasmonic nanostructures through electromagnetic field enhancement, achieving amplification factors of up to 10^11^. In EV diagnostics, SERS is used primarily in labeled (SERS-tag) or label-free modes. SERS tags use Raman reporter molecules conjugated to nanoparticles for targeted quantification, whereas label-free SERS captures intrinsic fingerprints of EV lipids and proteins. In SCI liquid biopsies, where neural EVs may be sparse, SERS offers high sensitivity and multiplexing capability because of narrow spectral bandwidths. Label-free modes enable noninvasive fingerprint characterization. However, signal reproducibility and substrate uniformity remain challenging, often requiring multivariate statistical analysis for accurate classification. To address EV heterogeneity, biomimetic substrates such as 3D gold-coated macroporous inverse opal structures have been engineered to entrap EVs and amplify signals via slow-light effects, enabling discrimination of disease-associated EVs based on phosphoprotein peaks. Although promising, SERS remains largely in the proof-of-concept phase for SCI and requires further substrate standardization to reduce signal variability [29,31].

Surface plasmon resonance (SPR): SPR monitors biomolecular interactions via refractive index changes near metal surfaces. Because individual EVs have low effective mass, nanoplasmonic approaches often use transmission SPR through periodic nanoholes or signal amplification strategies. SPR enables real-time, label-free monitoring of binding kinetics. Conventional SPR often lacks sensitivity for low-abundance EVs; however, nanoplasmonic designs improve field confinement, although nonspecific binding in complex biofluids remains a limitation. Nano-plasmonic sensors using periodic nanohole arrays confine electromagnetic fields to depths that match EV dimensions (<200 nm), enhancing sensitivity by up to 10^4^-fold compared with Western blotting. Further sensitivity can be achieved using catalytic nanomaterials. For example, reformative tyramine-based signal amplification can form DNAzymes on EV surfaces that catalyze deposition of gold nanoparticles, producing a geometrically enhanced SPR response. These nano-plasmonic approaches are transitioning from proof-of-concept to validation and demonstrate high-throughput profiling capabilities relevant to clinical workflows [32,33].

Colorimetry: Colorimetric biosensors transduce recognition events into visible chromatic shifts, typically through aggregation of plasmonic nanoparticles such as gold nanoparticles (AuNPs) or nanozyme-mediated catalysis. These assays facilitate point-of-care testing in acute trauma settings because of their simplicity and instrument-free readout. However, they generally exhibit lower sensitivity than fluorescence-based methods or SERS and are susceptible to background interference in complex matrices. In common aptamer-based systems, specific binding between EV surface proteins (e.g., CD63) and aptamers displaces the aptamers from AuNPs, triggering salt-induced aggregation and a red-to-blue color shift. To improve multiplexing, multidimensional color-coded nanoprobes targeting multiple proteins have been developed, with computer vision used to decode mixed-color fingerprints for disease subtyping. Although commercially attractive because of their simplicity, colorimetric assays are generally best suited as rapid screening tools rather than definitive quantitative diagnostics [30,34,35].

Immunochromatographic assay (ICA): Lateral flow immunoassays (LFIAs), also termed ICAs, are paper-based platforms that rely on capillary action to move samples across zones functionalized for sandwich immune-complex formation. Detection typically uses colloidal gold or fluorescent nanocomposites. ICAs are rapid (<20 min) and user-friendly, making them well suited for point-of-care settings. However, sensitivity is often limited by high background noise in blood or CSF after injury, necessitating signal amplification strategies for low-abundance EV markers. A double-gold nanoparticle conjugate system has been engineered to enhance sensitivity by approximately 13-fold by using a secondary AuNP conjugate to bind the primary EV-recognition conjugate. In addition, dual-signal LFIAs using core/dual-quantum dot shells provide both colorimetric and fluorescent readouts and can achieve limits of detection lower than standard enzyme-linked immunosorbent assays for some analytes. These platforms are well validated for other biomarkers and are being adapted for EV detection in point-of-care trauma scenarios [35,36,37].

Electrochemiluminescence (ECL): ECL assays detect light emission generated by chemical reactions or electron transfer at electrode surfaces. Nanomaterials can serve as carriers for signaling molecules (e.g., luminol) or as catalysts that amplify reactive species generation. ECL offers high sensitivity, low background, and wide dynamic range; however, these methods often require precise fluidic control and specialized instrumentation. An ultrasensitive ECL biosensor has been developed using dual amplification combining DNA barcodes and hybridization chain reaction (HCR). After EV capture, released barcode DNA triggers HCR to form long DNA duplexes that intercalate hemin, thereby substantially enhancing ECL signal. In addition, distance-dependent regulation of ECL using ferrocene-labeled aptamers enables signal-on or signal-off switching based on proximity to the luminophore. This distance-dependent mechanism can help filter nonspecific electrochemical signals common in ion-rich biofluids such as CSF. Although highly sensitive, these methods typically require sophisticated instrumentation, positioning them as validation tools rather than frontline point-of-care devices [38,39].

Fluorescence: Fluorescence biosensing detects EVs via fluorophore emission. Strategies to improve signal-to-noise ratios include fluorescence resonance energy transfer (FRET), plasmon-enhanced fluorescence (PEF), and target-triggered DNA circuits. Fluorescence-based approaches are highly sensitive and compatible with standard equipment. However, photobleaching and autofluorescence in biological fluids remain important limitations, particularly in blood-contaminated CSF or hemolyzed plasma after trauma. PEF using aluminum nanohole arrays has been reported to amplify signals from single EVs, enabling detection of low-expressing phenotypes that may be missed by conventional methods. FRET platforms using two-dimensional nanomaterials such as MXenes or graphene oxide can quench fluorophore-labeled aptamers; upon EV binding, the aptamers are released and fluorescence is restored for sensitive profiling. In addition, bivalent cholesterol anchors have been used to insert into EV membranes and trigger enzyme-free DNA circuits, enabling high-fidelity signal amplification [40,41,42].

Collectively, advances in EV isolation and detection technologies establish the essential pre-analytical foundation for reliable biomarker research in SCI. Accurate separation of vesicular from non-vesicular components and reproducible detection of EV subpopulations are critical prerequisites before meaningful molecular interpretation can occur. Building upon this methodological framework, subsequent sections focus on the biological significance of EV-associated cargos and their diagnostic and prognostic implications in SCI.

## 3. EV Dynamics After SCI

**Figure 3:**
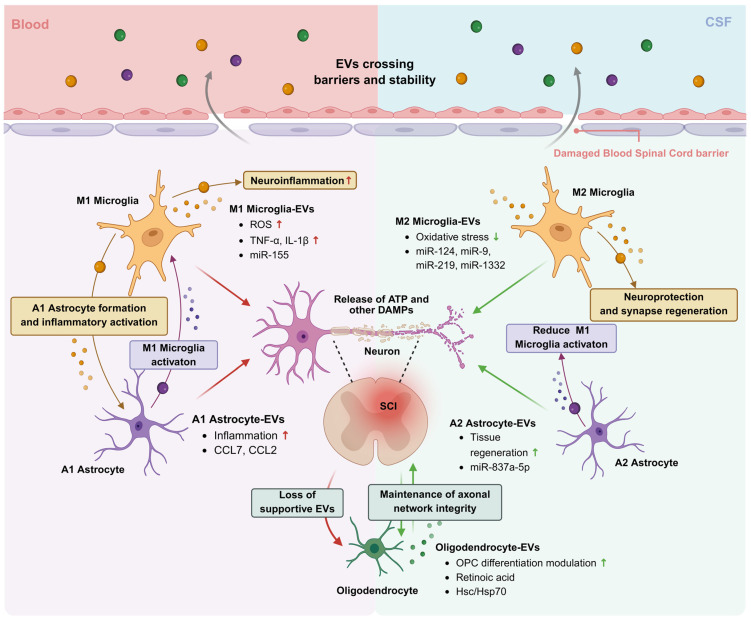
EV-mediated glial–neuronal communication after spinal cord injury. Following SCI, neurons release ATP and damage-associated molecular patterns (DAMPs), activating surrounding glial populations. Pro-inflammatory EVs derived from M1 microglia and A1 astrocytes carry cytokines (TNF-α, IL-1β) and miRNAs such as miR-155, amplifying secondary neuroinflammation. In contrast, EVs from M2 microglia and A2 astrocytes are enriched in reparative miRNAs (miR-124, miR-219, miR-873a-5p) that support neuroprotection and regeneration. Oligodendrocyte-lineage EVs provide metabolic and myelin-associated support (e.g., HSP70), although oligodendrocyte loss reduces axonal stability. Together, these EV-associated cargoes represent candidate biomarker signatures reflecting the balance between degeneration and repair. Abbreviations: ATP, adenosine triphosphate; DAMPs, damage-associated molecular patterns; ROS, reactive oxygen species; OPCs, oligodendrocyte precursor cells; EVs, extracellular vesicles; SCI, spinal cord injury.

EVs are increasingly recognized not only as mediators of intercellular communication after SCI but also as carriers of molecular signatures that reflect injury severity, inflammatory progression, and recovery potential. Although SCI pathophysiology involves complex neuroimmune interactions among neurons, glial cells, and peripheral immune compartments, this section highlights mechanistic pathways specifically in the context of EV-associated biomarker discovery. Rather than providing an exhaustive review of secondary injury mechanisms, we focus on EV-driven processes that directly shape the composition of circulating EV cargo, including inflammatory proteins, cytokines, and regulatory miRNAs with diagnostic and prognostic relevance. In particular, EV-mediated crosstalk among microglia, astrocytes, oligodendroglia, and neurons contributes to distinct molecular patterns that may be detected through liquid biopsy approaches. Moreover, bidirectional communication between the CNS and peripheral organs further influences EV profiles in blood, underscoring both their biomarker potential and the challenges of interpreting cellular origin under systemic inflammation. Overall, understanding these EV-linked mechanisms provides a biological rationale for the candidate biomarkers discussed in subsequent sections (Figure 3).

### 3.1. EVs Contribute to Local and Systemic Crosstalk After Neurotrauma, Including SCI

#### 3.1.1. Microglia–Neuron Communication

Failure of axonal regeneration after SCI is driven by multiple mechanisms, including inadequate chemoattraction, glial scar formation, and the presence of inhibitory molecules. Within this complex microenvironment, EVs act as critical mediators of local and systemic crosstalk between neurons and microglia [43,44]. Neurons also respond by secreting EVs that modulate microglial activity. Microglia-derived EVs can be protective or harmful, depending on whether microglia adopt an M1 or M2 phenotype. This aligns with the Yin–Yang model of microglia-derived EVs after neurological injury, in which EVs from pro-inflammatory microglia (Yin) carry harmful cargo and exacerbate tissue damage, whereas EVs from anti-inflammatory microglia (Yang) contain protective factors that promote recovery [45].

Under inflammatory conditions and neurological injury, microglia-derived EVs are enriched in harmful factors such as the let-7b/high mobility group box 1 complex and reactive oxygen species (ROS), which contribute to neuronal death, reduced neurite outgrowth, and increased apoptosis in PC12 neurons [46]. This enrichment is considered an initial trigger for altered EV dynamics after SCI. Subsequently, ATP released from injured neurons activates the P2X purinoceptor 7 receptor on microglia, triggering inflammasome activation and loading of interleukin (IL)-1β into EVs. Under these conditions, microglia activate nuclear factor kappa-light-chain-enhancer of activated B cells (NF-κB) signaling via lipopolysaccharide or TNF-α, and the released EVs carry miR-155, TNF-α, and glutaminase [47]. These acute-phase EV signatures may serve as molecular indicators of early neuroinflammatory severity and secondary injury progression after SCI.

Importantly, the microglial response is not entirely detrimental. M2 microglia-derived EVs exhibit neuroprotective properties by carrying miR-124 and anti-inflammatory mediators, thereby increasing neuronal survival and reducing inflammation [48]. miR-124 in microglial EVs, when taken up by neurons, inhibits ubiquitin-specific peptidase 14, a proteasome-regulating protein, thereby reducing oxidative stress and enhancing neuronal survival. Other miRNAs, including miR-9, miR-1332, miR-218, and miR-219, have also been implicated in neuroprotection and synapse regeneration [48]. This represents the Yang aspect of microglial EVs, in which the M2 phenotype dominates and EV cargo shifts toward anti-inflammatory and tissue-regenerative functions, counterbalancing the harmful effects of Yin-phase EVs. In this context, EV-associated miR-124 may serve as a biomarker candidate indicative of reparative immune modulation.

Microglia may represent an important source of EVs at the injury site; however, attributing the precise cellular origin of circulating EVs detected in blood remains challenging under systemic inflammation. EVs derived from microglia are enriched in miR-152-3p after SCI. When taken up by hippocampal neural stem cells, miR-152-3p inhibits WNT10b, leading to reduced Wnt/β-catenin signaling. This impairs hippocampal neurogenesis and results in defective neuronal differentiation and cognitive deficits [49]. These findings further suggest that microglial EV cargo may reflect not only local injury responses but also broader neurological sequelae relevant to biomarker interpretation.

#### 3.1.2. Astrocyte-Neuron Communication

After SCI, injured neurons release large amounts of ATP through pannexin-1 and connexin-43 hemichannels. This ATP surge acts as a critical signal that activates astrocytes via P2X7 receptors [50]. ATP, together with glutamate and ROS released from necrotic neurons, drives astrocytes into a reactive state and enhances EV secretion. Neuron-derived signals not only activate astrocytes but also influence the composition of astrocyte-derived EV cargo.

The inflammatory environment induces astrocytes to produce EVs with neurotoxic Yin characteristics, whereas other neuron-derived signals, such as fibroblast growth factor 1, promote production of protective heat shock protein 70/heat shock cognate 70 (HSP70/HSC70) EVs [51]. Thus, neurons are not passive during the acute phase of SCI but act as key initiators that regulate astrocyte responses and direct formation of two opposing EV populations: neurotoxic and neuroprotective.

In the acute inflammatory environment after SCI, astrocytes shift strongly toward the A1 state and begin releasing neurotoxic EVs. A notable feature is emergence of chemokine-enriched EV cargo, such as chemokine (C-C motif) ligand 7 (CCL7)-containing EVs, in which the EV membrane protects CCL7 from degradation. This prolongs bioactivity and amplifies inflammatory responses through activation of M1 microglia [52]. Concurrently, astrocytes release CCL2-enriched small EVs that exert dual effects on microglia and neurons: they activate microglia through C-C chemokine receptor type 2 (CCR2) and induce apoptosis in CCR2-positive neurons [53]. Collectively, these neurotoxic EV signatures may reflect acute secondary inflammatory amplification and injury evolution after SCI.

Alongside neurotoxic signals, astrocytes after SCI also generate protective EV populations, often associated with the Yang state. One prominent mechanism is the release of HSP70/HSC70 EVs, which can reduce intracellular stress and protect neurons from apoptosis [51]. In addition, astrocytes release vimentin-containing EVs, which appear after mechanical injury or inflammation and may enhance axonal regeneration [54]. Finally, a subset of EVs carrying hepaCAM and miR-873a-5p can reduce microglial activation and promote neurite outgrowth, contributing to stabilization of the neural microenvironment and supporting tissue regeneration after injury [52]. In this context, astrocyte-associated EV cargo may serve as a biomarker indicator of the balance between neurotoxic inflammation and endogenous repair responses.

After SCI, astrocytes, microglia, and neurons form a multidirectional signaling network in which EVs play a central role. Microglia are potent activators after injury; they release TNF-α and C1q, which promote astrocyte transition to the reactive Yin state and shift astrocyte-derived EV cargo toward more pro-inflammatory profiles [52,54]. Conversely, astrocytes also send EVs back to regulate microglia. Certain EV populations released under Yang-biased conditions can reduce microglial activation, thereby limiting propagation of inflammatory responses. Neurons participate in this network via feedback signals. When stressed or degenerating, neurons release ATP and DAMPs, sustaining astrocyte and microglial activation and increasing EV secretion [53]. These bidirectional EV-mediated interactions provide a mechanistic basis for interpreting circulating EV biomarkers in SCI, although assigning precise CNS cellular origins remains challenging under systemic inflammation.

#### 3.1.3. Oligodendroglia-Neuron Communication

Following SCI, axonal damage is accompanied by increased glutamate release and neuronal stress signaling, creating a wave of activation that propagates to oligodendrocyte precursor cells (OPCs) and mature oligodendrocytes. Glutamate-driven excitotoxic stress promotes activity-dependent EV release from oligodendrocytes, linking axonal injury to demyelination dynamics [55]. Beyond glutamate signaling, neurons also regulate the oligodendrocyte lineage through EVs that carry retinoic acid, which modulates retinoic acid receptor signaling in OPCs. Neuron-derived EVs can either promote or inhibit OPC differentiation depending on the microenvironment, thereby influencing the myelin regeneration process [56].

Oligodendrocytes respond to neuronal activation by releasing a population of EVs that directly support axons. These EVs contain HSC/HSP70, metabolic enzymes, and myelin-specific proteins, enabling multilayered protection of neurons [55]. Such myelin- and chaperone-associated EV cargo may therefore represent candidate biomarkers reflecting demyelination severity and axonal vulnerability after SCI.

Notably, uptake of oligodendrocyte-derived EVs occurs primarily in the periaxonal region, at the junction between myelin and the axon. Although oligodendrocytes support neurons through EV secretion, they are highly vulnerable after SCI. Excess glutamate at the injury site causes Ca^2+^ overload, disrupts cellular homeostasis, and renders both oligodendrocytes and OPCs prone to stress and degeneration [55]. When oligodendrocytes are lost, production of axon-supportive EVs decreases substantially, increasing the risk of secondary degeneration due to insufficient metabolic and chaperone support.

In addition to loss of mature oligodendrocytes, OPC regulation is disrupted after SCI. Retinoic acid signaling from neurons, which helps regulate OPC differentiation, becomes unbalanced, and EVs associated with retinoic acid may be released asynchronously, thereby hindering coordinated myelin regeneration [56]. These disruptions further suggest that oligodendrocyte-derived EV signatures could provide mechanistic insight into chronic neurodegeneration and impaired repair.

Within injured spinal cord tissue, oligodendrocyte-derived EVs also interact with microglia. A substantial portion of EVs released from the oligodendrocyte lineage may be internalized by activated microglia, limiting delivery of supportive EV cargo to neurons during the acute inflammatory phase [55]. Consequently, reduced EV cargo enriched in myelin proteins and chaperones may serve as a molecular indicator of failed metabolic support and progressive demyelination after SCI.

### 3.2. Bidirectional CNS–Peripheral Crosstalk Mediated by EVs

#### 3.2.1. Acute Phase Response Modulation

CNS injuries, including SCI and TBI, trigger not only a localized inflammatory reaction at the injury site but also a systemic acute phase response (APR) characterized by a marked increase in cytokines, acute phase proteins, and systemic immune dysregulation. Growing evidence suggests that the APR is not simply a secondary consequence of tissue inflammation but is actively driven by signals originating from injured neural tissue. Among these signals, EVs appear to be a key mediator linking CNS inflammation to systemic activation because they can transport inflammatory molecules, RNAs, and damage-associated molecular patterns (DAMPs) from the injury site into the circulation [3].

Mechanistic studies show that EVs can migrate to the liver and spleen, where they activate NF-κB-dependent signaling pathways, promote cytokine production, and modulate immune cell kinetics at the systemic level [57]. Following SCI, blood–brain barrier leakage facilitates the entry of CNS-derived EVs into the circulation. Importantly, these CNS-derived EV cargo profiles may serve as early circulating indicators of injury-triggered systemic inflammation. At a mechanistic level, neuroendothelial-derived EVs represent one of the earliest and strongest signals transmitted from the CNS to the liver. These EVs are preferentially taken up by Kupffer cells and promote polarization toward a pro-inflammatory phenotype. This shift increases TNF-α and CXCL1 production and promotes neutrophil recruitment, thereby initiating the APR [58]. Notably, the EV fraction alone—even in the absence of free cytokines—is sufficient to trigger this response, indicating that EVs can function as active signaling units rather than passive carriers.

Astrocyte-derived EVs also modulate the liver’s inflammatory threshold. Under neuroinflammatory conditions, these EVs inhibit peroxisome proliferator-activated receptor-α, thereby removing metabolic suppression of NF-κB signaling and enhancing acute-phase cytokine production [59]. This observation suggests that systemic inflammatory biomarkers measured in blood may partly reflect EV-mediated immune reprogramming rather than local spinal pathology alone.

Together, these EV pathways illustrate that the APR is actively initiated by CNS-derived signals, while peripheral organs subsequently amplify and redistribute inflammatory cues. Once activated, the liver and immune system become secondary signal sources that feed back to influence the CNS. EVs originating from the spleen, monocytes, and neutrophils can carry cytokines and immune-regulatory RNAs, increasing vascular permeability and promoting leukocyte migration into neural tissue [57,59]. This bidirectional feedback underscores a major translational challenge: circulating EV signatures after SCI may represent a composite of CNS injury signals and systemic immune confounders.

#### 3.2.2. Coagulation and Vascular Effects

SCI causes profound disruption of vascular endothelial function at the injury site and throughout the circulatory system. When the endothelium is activated or damaged, endothelial cells release large amounts of EVs into the blood. These EVs carry markers reflecting endothelial inflammation along with signals capable of promoting hypercoagulability. In preclinical studies, endothelial EV levels increase markedly immediately after injury, indicating that EV release is among the earliest vascular responses to SCI [60]. These EVs expose phosphatidylserine and may carry tissue factor, thereby promoting thrombin generation and acute hypercoagulability. In addition, endothelial EVs contain adhesion molecules such as ICAM-1 and VCAM-1, contributing to leukocyte adhesion and vascular inflammation. Together, these endothelial EV-associated components may serve as early circulating biomarkers of thrombo-inflammatory activation after SCI [3,60,61].

In addition to endothelial EVs, platelets represent another major EV source in the acute phase. Platelet-derived EVs are enriched in phosphatidylserine, platelet factor 4 (PF4), thrombospondin-1, and chemokines such as CCL5, enhancing platelet–immune cell interactions and creating conditions for microthrombus formation. Notably, elevated platelet EV levels may persist after SCI and have been linked to increased risk of deep vein thrombosis and hematological complications in patients [12,62]. Thus, platelet EV cargo may provide prognostic indicators for vascular complications beyond the primary spinal injury.

The CNS is also a significant source of EV-mediated signals that influence vascular function. EVs originating from astrocytes and microglia carry miRNAs that regulate endothelial tight junction proteins. miRNAs such as miR-155, miR-21, and miR-146a can reduce expression of occludin, claudin-5, and zonula occludens-1, thereby decreasing integrity of the blood–spinal cord barrier and increasing vascular permeability. These EV-associated regulatory signals may contribute to blood–spinal cord barrier disruption and secondary ischemic vulnerability [3,62,63,64].

Thus, EVs from the endothelium, platelets, and CNS collectively form a complex signaling network that impairs vascular function after SCI, and specific molecules within these EVs are decisive mediators of these processes.

### 3.3. Spinal Cord Injury Alters Circulating EV Count and miRNA Cargo

Circulating EVs in blood change in a subtype-specific manner after SCI, depending on the vesicle population analyzed. At an early stage, the total amount of EVs in plasma isolated by ultracentrifugation decreases significantly at one day post-injury, suggesting that the initial circulatory response to SCI may involve a general reduction in total circulating EVs. In contrast, CD81-positive EVs increase markedly at the same time point and remain elevated through day seven. This pattern indicates that SCI alters not only EV quantity but also the distribution of EV subpopulations, with preferential enrichment of CD81-positive vesicles [65]. These temporal changes may provide an early biomarker window reflecting acute injury-driven EV release dynamics.

Alongside quantitative shifts, circulating EV cargo undergoes substantial molecular remodeling after SCI. In mouse models, serum EVs show enrichment of pro-inflammatory and cell death-related miRNAs as early as six hours post-injury, including miR-152-3p, miR-130a-3p, and miR-126a-5p. These increased miRNAs are predicted to participate in inflammatory signaling pathways, mitogen-activated protein kinase (MAPK) signaling, stress responses, and apoptosis, reflecting early activation of secondary injury processes. Conversely, synapse-associated miRNAs such as miR-125b-5p decrease sharply during the same period, indicating that SCI rapidly reshapes EV cargo toward inflammatory signaling while reducing neuroprotective components [66].

In the subacute phase, miRNA changes in EVs persist but display distinct temporal characteristics. Multiple pro-inflammatory miRNAs among the reported panel of 65 neural miRNAs increase in plasma EVs from day one to day seven after SCI, whereas some anti-inflammatory miRNAs decrease. Although the complete list of altered miRNAs has not been fully reported, available data indicate sustained elevation of several pro-inflammatory miRNAs during this interval. This pattern is consistent with continued increases in CD81-positive EVs in plasma over several days after injury and may further enhance inflammatory signaling between the central and peripheral nervous systems [65].

Beyond in vivo models, inflammatory stimulation of cultured astrocytes is sufficient to alter EV content. When astrocytes are treated with IL-1β or TNF-α, EV numbers increase, and miR-125a-5p and miR-16-5p become enriched in secreted EVs, potentially affecting neuronal survival and axonal branching. However, translation of such in vitro signatures to clinical biofluids requires cautious interpretation [67].

Non-miRNA EV cargo has also been reported to change after SCI. Syntheses of clinical and preclinical studies indicate that EVs isolated from plasma of SCI patients show increased abundance of lipid-related and inflammatory proteins such as apolipoproteins, HSP70, and S100A8/A9. Concurrently, surface tetraspanins such as CD9, CD63, and CD81 change over time, suggesting that the injury environment influences the origin and phenotype of circulating EV populations. In addition, increased endothelial- and platelet-derived EVs in the early phase correlate with injury severity, supporting EVs as markers of vascular activation and coagulation responses after SCI [9].

Recent studies indicate that EV surface epitopes in plasma bear distinct signatures after SCI and may reflect the origin and activation state of secretory cells. In trauma cohorts, multiplex analysis of EV surface markers identified epitopes capable of distinguishing SCI from other types of trauma. Markers such as CD13, CD196, myelin oligodendrocyte glycoprotein, CD133, and myelin basic protein were more prevalent in EV populations from individuals with neurological injury. These findings suggest that plasma EVs may carry markers of neurons, glial cells, or immune cells involved in systemic inflammatory responses. Accordingly, analysis of EV surface epitopes may provide valuable information about tissue injury status and immune activity in SCI patients.

In animal models of SCI, EV surface epitope profiles evolve over time and depend on injury severity. Platelet- and endothelial-related epitopes such as CD41, CD44, and CD61 also increase within the CD9-positive subpopulation during early stages. This pattern highlights contributions of vascular responses and platelet activation to EV population dynamics after SCI. In the chronic phase, EV numbers decrease compared with the early phase, reflecting the transition of inflammatory and hemodynamic responses over the course of recovery [68].

Overall, SCI-associated EV alterations represent a selective and time-dependent process involving changes in EV abundance, inflammatory miRNA enrichment, and evolving surface marker profiles. These circulating EV signatures may support diagnosis, stratification, and monitoring of injury progression, although systemic inflammation and comorbid trauma remain important confounders.

## 4. Biomarkers for Diagnosis, Prognosis, and Complication Management in SCI

Accurate stratification of injury severity, prediction of neurological recovery, and monitoring of secondary complications remain central challenges in the clinical management of SCI. While imaging modalities and neurological scales such as the ASIA Impairment Scale provide essential structural and functional information, they do not directly capture dynamic molecular changes occurring within the injured spinal cord. EVs, which transport proteins and regulatory RNAs across biological barriers, offer a minimally invasive window into these evolving pathological processes. Their molecular cargo reflects cellular stress, neuroinflammation, axonal injury, and repair responses, positioning EV-associated biomarkers as potential tools to complement conventional assessment strategies.

**Figure 4:**
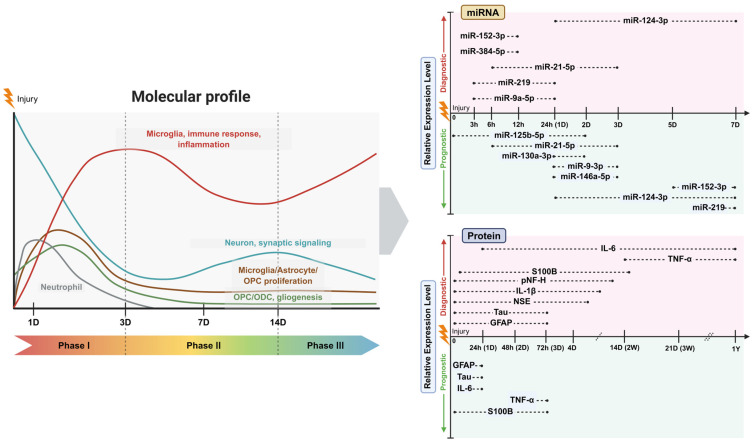
Temporal detection windows of EV-associated miRNAs and protein biomarkers after spinal cord injury. This schematic summarizes reported post-injury time ranges during which candidate EV-miRNAs and classical protein biomarkers are detectable in blood or CSF. Early-phase markers (hours–days) are primarily associated with injury severity stratification (diagnostic utility), whereas later or sustained signals (days–weeks) correlate with neurological recovery, demyelination, or secondary injury progression (prognostic utility). EV-miRNAs (top), including miR-9-3p, miR-21-5p, miR-219, and exploratory preclinical candidates (e.g., miR-152-3p, miR-384-5p), show distinct phase-dependent kinetics. Protein biomarkers (bottom), such as S100 calcium-binding protein B (S100B), glial fibrillary acidic protein (GFAP), neuron-specific enolase (NSE), Tau (microtubule-associated protein tau), phosphorylated neurofilament heavy chain (pNF-H), and interleukin-6 (IL-6), provide complementary temporal profiles. Together, these timelines highlight the potential value of integrating EV-miRNA panels with established protein markers for American Spinal Injury Association Impairment Scale (AIS) stratification and outcome prediction. Dotted lines indicate detection windows reported across studies (not peak expression). Abbreviations: S100B, S100 calcium-binding protein B; GFAP, glial fibrillary acidic protein; NSE, neuron-specific enolase; Tau, microtubule-associated protein tau; pNF-H, phosphorylated neurofilament heavy chain; IL-6, interleukin-6; TNF-α, tumor necrosis factor-alpha.

The temporal resolution offered by EVs further supports their conceptual relevance for clinical application. In the acute phase of SCI, rapid changes in EV-associated stress signals and inflammatory mediators may reflect early tissue damage and injury severity As pathology progresses into the subacute phase, EV cargo profiles often shift toward mediators involved in neurovascular remodeling and tissue repair, potentially providing insight into regenerative capacity and secondary injury cascades. In the chronic phase, persistent fibrotic or inflammatory signatures may correspond to maladaptive sequelae, including glial scarring or neuropathic pain. Although currently reported temporal windows largely reflect sampling intervals rather than validated kinetic peaks, phase-specific EV profiling provides a framework for future longitudinal monitoring and molecularly informed therapeutic strategies (Figure 4) [13,69,70].

### 4.1. Protein Markers of Neural Injury Response

#### 4.1.1. Glial and Neuronal Structural Proteins as Diagnostic Classifiers

Effective recovery following spinal cord interventions depends on early recognition of neurological changes and precise phenotyping of patient responses. In this regard, EV-based biomarkers offer a promising noninvasive tool for monitoring phenotypic shifts and managing potential complications. Quantification of structural proteins released after cellular necrosis or membrane disruption has emerged as a robust approach for objectively stratifying injury severity, particularly when American Spinal Injury Association Impairment Scale (AIS) grading is difficult to ascertain (Figure 4) [71].

S100 calcium-binding protein B (S100B): Predominantly localized in astroglia and Schwann cells, S100B is a well-characterized marker of glial injury. Clinical studies in human cohorts have shown that serum and CSF S100B levels rise rapidly after acute SCI and correlate with injury severity. In a prospective study of 60 acute SCI patients, serum S100B peaked on day 4 and was significantly higher in motor-complete (AIS A/B) than in incomplete (AIS C/D) injuries. Similarly, CSF S100B measured at 24 h distinguished AIS grades in a 50-patient cohort, supporting its value as a severity biomarker. However, S100B has a short serum half-life (~24 h), and peripheral measurements often lack specificity because extracranial sources, including adipose tissue and bone fractures, can also release S100B. In contrast, isolating astrocyte-derived EVs carrying S100B may reduce this background noise and provide a more CNS-specific indicator of glial injury than free serum S100B [72,73,74,75].

Glial fibrillary acidic protein (GFAP): As an intermediate filament protein specific to astrocytes, GFAP serves as an indicator of astroglial injury and reactive gliosis. In a cohort of 35 acute SCI patients, serum GFAP levels were significantly higher in severe (AIS A) injuries within the first 24 h. A combined CSF biomarker model incorporating GFAP, S100B, and IL-8 predicted baseline AIS grade with 89% accuracy. Unlike S100B, GFAP is highly CNS-specific; however, serum detection depends on disruption of the blood–spinal cord barrier. Because EVs can cross the blood–spinal cord barrier via transcytosis, GFAP-positive EVs may provide a more consistent marker of astrogliosis, particularly in chronic phases when barrier permeability is reduced [76,77,78].

Neuron-specific enolase (NSE): NSE is a glycolytic enzyme localized in the cytoplasm of central neurons and is used as a marker of neuronal cell body injury. In serum, NSE shows rapid kinetics, peaking within 12–48 h post-injury and then declining sharply. Although NSE has been reported to predict prognosis with a sensitivity of 74.35%, it may be less effective than S100B or GFAP for distinguishing AIS grades. The short diagnostic window of free NSE may be addressed by EV-encapsulated NSE, which could be protected from degradation and potentially enable subacute monitoring of ongoing neuronal apoptosis [72,73,74,75,78].

Phosphorylated neurofilament heavy subunit (pNF-H): pNF-H is a major cytoskeletal component of large myelinated axons. In a pilot study of 14 cervical SCI patients, plasma pNF-H displayed delayed kinetics, becoming detectable at 12 h and remaining elevated for up to 21 days, unlike the rapid decline of S100B and NSE. Levels in AIS A patients were 10–100-fold higher than in incomplete injuries, supporting pNF-H as a marker of cumulative axonal loss. Its sustained elevation suggests ongoing axonal breakdown, and EV-associated pNF-H could potentially reflect Wallerian degeneration and serve as a biomarker of white matter tract integrity that is less dependent on gray matter injury [72,79].

Tau: Tau is a microtubule-associated protein enriched in neurons and axons. In a prospective cohort of 16 patients, CSF Tau levels were significantly higher in motor-complete than in incomplete injuries, and elevated early Tau correlated with poorer motor recovery at 6 months. Because Tau is susceptible to aggregation and proteolytic cleavage, assessment of EV-associated Tau may help distinguish pathological neurodegenerative release from physiological clearance and may provide insight into post-traumatic neurodegeneration after SCI [74,76,77].

#### 4.1.2. Inflammatory Cytokines and the Secondary Injury Cascade

Secondary injury after SCI is driven largely by neuroinflammation. Temporal expression patterns of pro- and anti-inflammatory cytokines provide insight into the evolving molecular phenotype of injury.

IL-6: IL-6 is a pleiotropic cytokine that is rapidly upregulated following trauma. In human SCI, CSF IL-6 levels can reach orders of magnitude higher than serum concentrations. Elevated IL-6 at 24 h is a strong predictor of injury severity and correlates with motor recovery. The massive discrepancy between CSF and serum IL-6 levels suggests limited spillover into the periphery. EVs could act as shuttles, transporting high concentrations of cytokines like IL-6 from the lesion site to the periphery. Isolating cytokine-loaded EVs could provide a liquid biopsy of the neuroinflammatory niche without the need for invasive lumbar punctures [76,77,80].

TNF-α and IL-1β: These pro-inflammatory cytokines initiate the secondary injury cascade. In human histological studies, immunoreactivity for IL-1β and TNF-α is detected in neurons within 0.5 h of injury. Serum studies show temporal fluctuations, with TNF-α levels significantly elevated in chronic SCI patients compared with controls. However, detection in CSF is often inconsistent because of assay sensitivity limitations. The difficulty in detecting IL-1β and TNF-α in CSF using standard multiplex assays highlights an opportunity for EV research. Cytokines encapsulated in EVs may be concentrated to detectable levels, offering a more sensitive readout of neuroinflammation than free-fluid analysis. In addition, membrane-bound TNF-α on EVs may have distinct signaling properties compared with soluble TNF-α [77,80,81,82,83].

IL-10: IL-10 is an anti-inflammatory cytokine of interest because of its neuroprotective potential. However, in cross-sectional studies of chronic SCI patients, serum IL-10 was largely undetectable, and no significant differences were observed between SCI patients and controls. This low detectability in peripheral blood highlights the challenge of using IL-10 as a circulating biomarker without enrichment strategies such as EV isolation [83].

While the prognostic utility of free-circulating structural proteins and inflammatory cytokines in CSF and serum is well documented, translation into robust clinical biomarkers is often limited by kinetic instability, rapid clearance, and dilution upon entry into peripheral circulation [73]. For example, transient expression of pro-inflammatory mediators such as IL-1β and TNF-α, which often decline to undetectable levels shortly after the primary injury, imposes a narrow temporal window for diagnostic capture using standard immunoassays [84].

Furthermore, the substantial concentration gradient between CSF and serum for markers such as IL-6 suggests that transmission of free proteins across the blood–spinal cord barrier is inefficient and/or subject to rapid systemic dilution [77,80]. In this context, EVs have been postulated to serve as stable biological vehicles that protect labile cargo from proteolytic degradation and potentially facilitate transport across the blood–spinal cord barrier, thereby preserving molecular fidelity of CNS lesions within a peripheral liquid biopsy [13,85]. Consequently, the relative stability of EV-associated cargo could refine biological stratification of injury severity, potentially distinguishing between patients with complete injuries (AIS A) who have neuroplastic potential for AIS grade conversion and those who do not [76,86].

However, this hypothesis should be approached with caution. Although sequestration of targets such as S100B, GFAP, and pNF-H within EVs is theoretically advantageous for enhancing diagnostic sensitivity and extending diagnostic windows beyond rapid declines observed in free forms, robust empirical validation of these specific protein cargos within SCI-derived EVs remains necessary before definitive clinical claims regarding superiority over total protein analysis can be established [72].

### 4.2. Translational Potential of miRNA Biomarkers for Injury Severity Assessment and Neurological Outcome Prediction

miRNAs have two key characteristics—tissue specificity and stability in biological fluids—that support their potential as biomarkers. Changes in miRNA levels after SCI are a prerequisite for their diagnostic and prognostic utility. Currently, the clinical standards for SCI diagnosis are MRI and the ASIA disability rating scale; however, poor baseline condition, concomitant injuries, and limited access to medications and surgical treatment can reduce reliability of these assessments [87]. Proteins are sometimes measured in CSF to determine injury location and extent, but CSF sampling requires an invasive spinal tap that is technically challenging and has low patient acceptance. Therefore, identification of biomarkers that can be obtained directly from blood or other accessible body fluids may be more useful for clinical practice and targeted interventions. Evidence indicates that miRNAs are tissue-specific and stable in body fluids, supporting their use as blood-based biomarkers [88]. A substantial body of evidence on serum and CSF miRNAs in human SCI suggests that early molecular injury signals are encoded in small RNA signatures. Because EVs are primary carriers of miRNAs in circulation, these findings provide mechanistic support for EV-associated miRNAs as early diagnostic biomarkers. To enhance interpretative clarity, candidate miRNAs discussed below are stratified into evidence tiers based on study design (human vs. preclinical), cohort size, presence of independent validation, and reproducibility across studies. Tier 1 includes biomarkers supported by human cohorts with relatively larger sample sizes or independent validation; Tier 2 represents preliminary human data with limited cohort sizes or without external validation; and Tier 3 comprises biomarkers currently supported primarily by animal or in vitro evidence. Notably, currently available human SCI studies generally involve modest cohort sizes (approximately 14–60 patients), underscoring the exploratory nature of the field and the need for larger validation studies (Figure 4).

#### 4.2.1. Acute-Phase Inflammatory miRNAs

The miRNAs in this cluster function as immediate molecular sentinels, reflecting the severity of primary mechanical trauma and initiation of secondary inflammatory cascades.

miR-384-5p: miR-384-5p, identified in murine serum and spinal cord tissue, has been proposed as a biomarker of injury severity and secondary damage in SCI. In a Sprague Dawley rat model, miR-384-5p was significantly elevated as early as 12 h after SCI, and the magnitude of increase correlated with injury severity. Compared with serum proteins such as S100B, NSE, or pNF-H, which show slower kinetics and lower discriminatory power, early and graded changes in miR-384-5p may provide an advantage [89]. Mechanistically, miR-384-5p regulates cellular stress responses. In dopaminergic neurons and spinal tissue, miR-384-5p targets the 3′-UTR of Beclin-1 and glucose-regulated protein 78. Downregulation in tissue (or efflux into serum) has been associated with activation of autophagy and endoplasmic reticulum stress pathways, contributing to neuronal apoptosis [90].

miR-152-3p: The molecular trajectory of miR-152-3p after SCI provides a characteristic signature that may reflect both acute pathogenesis and subsequent recovery mechanisms. In rat models, tissue levels reach a nadir at 128 h post-injury, whereas serum EV levels are significantly upregulated during the acute phase. This inverse relationship highlights a biofluid–tissue paradox but supports miR-152-3p as a candidate diagnostic marker for acute SCI. Mechanistically, miR-152-3p has been implicated in the regulation of c-Jun-related pathways involved in inflammation. In addition, EV-delivered miR-152-3p targets Wnt10b to suppress hippocampal neurogenesis, potentially linking spinal injury to remote cognitive complications [49,61,91,92,93].

miR-130a-3p: miR-130a-3p is a molecular indicator of neuroinflammation and may predict development of neuropathic pain (NP) after SCI. Unlike tissue downregulation observed for some protective miRNAs, miR-130a-3p expression is significantly upregulated in lumbar spinal tissue (L4–L5 dorsal horn) in SCI rats in a time-dependent manner. This signature is also reflected in circulation, as serum EV-associated miR-130a-3p levels are significantly increased in acute SCI, supporting its potential as a readily detectable biomarker of early injury severity and secondary inflammatory activation. Conservation of its sequence across humans, primates, and rodents supports translational relevance [92,93,94,95,96]. Mechanistically, miR-130a-3p targets and represses insulin-like growth factor-1, thereby activating NF-κB signaling and promoting microglial activation that contributes to hyperalgesia [94,95].

miR-9a-5p: miR-9a-5p, observed in rat spinal tissue and CSF, has been proposed as a marker of ischemic damage and blood–spinal cord barrier disruption. Its levels decrease significantly within 24–48 h post-injury. This downregulation may disinhibit MAP2K3 and Notch2, thereby exacerbating pro-inflammatory cytokine release (IL-6, IL-1β) and apoptosis and linking acute ischemia to secondary inflammatory cascades [97].

#### 4.2.2. Reparative and Regenerative miRNAs

These miRNAs are linked to specific cellular compartments—oligodendrocytes, neurons, and glia—and fluctuating levels may reflect CNS attempts at remyelination and repair.

miR-219: miR-219 is a CNS-enriched miRNA that is highly expressed in OPC and myelin lineages. Evidence suggests that miR-219 reflects myelin destruction, OPC dysfunction, neuroinflammation, and metabolic stress after SCI. In a mouse model of SCI, serum miR-219 levels increased markedly within 12 h after injury, and this increase correlated linearly with injury severity. Notably, this change was reported to be specific to the nervous system and not confounded by peripheral soft tissue injury. A proposed mechanism is that myelin destruction and OPC degeneration release miR-219 into circulation [89]. In contrast to serum, miR-219 decreases sharply in injured spinal cord tissue after SCI, reaching its lowest level on day 7. This decrease is accompanied by OPC proliferation, astrocyte activation, loss of mature OPCs, and expansion of demyelinated areas. The magnitude of miR-219 reduction correlates with histopathological injury, suggesting that local miR-219 levels reflect capacity for myelin preservation and endogenous repair [98]. Therefore, tissue-associated or EV-associated miR-219 may serve as a marker of white matter injury and a predictor of long-term neurological recovery. Mechanistically, miR-219 is essential for oligodendrocyte differentiation through repression of PDGFR and Sox6. Furthermore, EV delivery of miR-219-5p has been reported to alleviate neuronal ferroptosis via the UBE2Z/NRF2 pathway, promoting functional recovery [98,99,100].

miR-124-3p and miR-125b-5p: miR-124-3p, a brain-enriched miRNA, has potential as a neuronal injury marker. Detected in neuron-derived EVs in plasma, it correlates with neuronal lysis. Delivery of miR-124-3p via EVs has been reported to reduce neurotoxic glial activation and promote functional recovery. Its therapeutic mechanism is centered on immunomodulation: it regulates microglial polarization by targeting MYH9 and Ern1, suppressing the pro-inflammatory M1 phenotype and fostering a neuroprotective microenvironment important for subacute repair [93,101,102,103]. In contrast, miR-125b-5p may function as an inverse biomarker of injury. It is consistently downregulated in acute-phase tissue (days 3–14) and serum EVs, and reduced levels correlate with poor regenerative potential. Restoration of miR-125b-5p has been reported to support neurite outgrowth by targeting Sema4D (an axonal repulsion cue) and to modulate glial scar formation via MAPK signaling [93,104,105,106].

#### 4.2.3. Chronic-Phase/Complication-Related miRNAs

This category includes miRNAs with potential for predicting long-term functional recovery, fibrosis, and chronic immune dysregulation.

miR-9-3p: miR-9-3p is among the most extensively studied CNS-enriched miRNAs in neurotrauma. Evidence indicates that miR-9-3p is primarily expressed in the CNS. Multilayered analyses suggest that EVs containing miR-9-3p, which increases after SCI, originate largely from astrocytes in the brain rather than from blood or the injured spinal cord region. This interpretation is supported by data showing that miR-9-3p concentrations decrease at the lesion site but increase in the brain and in CSF EVs after SCI. After SCI, miR-9-3p increases significantly in CSF EVs from the acute phase onward. This increase has been interpreted as reflecting a neuroprotective response program initiated by the CNS to stabilize neural function. Notably, SCI patients with spontaneous neurological recovery had significantly higher EV-miR-9-3p levels, and discrimination of recovery versus non-recovery groups achieved an area under the curve (AUC) of 0.80 with a sensitivity of 80% and a specificity of 89%. This represents one of the strongest reported prognostic signals for an SCI-related miRNA to date. Mechanistically, EV-miR-9-3p may contribute to neuronal metabolic adaptation by suppressing energy-demanding pathways while stabilizing synaptic structure and promoting neuroplasticity. Neuroprotective effects of the miR-9 family have also been reported in spinal cord ischemia models, in which miR-9 reduces cytokine release, limits blood–spinal cord barrier disruption, and attenuates apoptosis through MAPK- and Notch2-related regulation [97,107].

Data from cerebral ischemia suggest that miR-9-3p is also a sensitive marker of acute neurological injury, and oxidative stress studies support stability of miR-9 as an early diagnostic indicator of neuronal injury [108,109]. Its neural origin, stability in EVs, mechanistic relevance, and clinical evidence of prognostic performance position miR-9-3p as a leading candidate for EV-based diagnostics in SCI.

miR-21-5p: miR-21-5p plays a prominent role in CNS injury and occupies a central position in networks regulating inflammation, cellular stress, and neuronal protection after SCI. In SCI patients, serum miR-21-5p levels increase markedly from the acute phase, and increases correlate with injury severity according to AIS classification. Levels also vary over time post-injury, indicating that miR-21-5p reflects both the extent of tissue destruction and the stage of disease progression. Preclinical data show that miR-21 increases markedly in the cerebral cortex and hippocampus 6–72 h after TBI and increases across multiple SCI models [110].

At the cellular level, miR-21-5p is upregulated in spinal neurons and exerts neuroprotective effects by suppressing pro-apoptotic pathways, reducing caspase-3 activation, and limiting neuronal loss. Inhibition of miR-21-5p worsens cell death and motor outcomes, whereas augmentation improves functional recovery [111]. When encapsulated in EVs, miR-21-5p exhibits enhanced stability and potential therapeutic relevance. EV-mediated miR-21 delivery has been reported to reduce apoptosis, decrease glial scar formation, and promote spinal cord regeneration, reflecting the intensity of endogenous repair responses after injury [112]. Immunologically, miR-21-5p may act as a bidirectional modulator, contributing to early inflammatory activation while supporting anti-inflammatory feedback in later stages. Its expression in immune cells, including T cells and monocytes/macrophages, further links CNS injury responses with systemic immune regulation [113].

miR-146a-5p: miR-146a-5p is a key regulator of innate immune signaling and neuroinflammation, making its expression profile relevant to SCI pathophysiology. In acute SCI animal models, endogenous miR-146a-5p expression is typically downregulated in spinal cord tissue, suggesting that the negative feedback mechanisms that normally constrain inflammation are compromised early in the disease process [114,115,116,117].

Despite endogenous deficiency, exogenous augmentation of miR-146a or miR-146a-5p has been reported to improve functional recovery, reduce inflammatory responses, and decrease neuronal apoptosis. Mechanistically, miR-146a-5p acts as a negative feedback modulator of Toll-like receptor/NF-κB signaling by targeting upstream effectors, including interleukin-1 receptor-associated kinase 1 and TNF receptor-associated factor 6. Repression of these targets reduces NF-κB activation and downstream transcription of pro-inflammatory cytokines such as IL-1β, IL-6, and TNF-α. In addition, miR-146a-5p targets G-protein-coupled receptor 17, and suppression of this receptor has been linked to reduced neuronal apoptosis and improved functional recovery. As a biomarker, miR-146a-5p is among the most abundant cargo miRNAs in hUCMSC-derived EVs, and delivery via modified EVs has been reported to suppress neurotoxic astrocyte markers, highlighting its role in modulating cellular components of the injury environment [93,114,115,116,117,118,119].

The current landscape of EV-derived miRNA research in SCI reveals a bifurcation between candidates supported by emerging human validation and those largely confined to preclinical exploration, each with distinct translational promise and limitations. Among the ten evaluated miRNAs, miR-9-3p, miR-21-5p, and miR-219 (particularly miR-219-5p) currently have the strongest human-associated evidence, positioning them at the forefront of clinical translation. Notably, miR-9-3p is a promising prognostic EV-miRNA candidate; high-throughput sequencing of human CSF EVs identified elevated miR-9-3p as a predictor of spontaneous functional recovery (AIS grade conversion), achieving an AUC of 0.80 [107]. Similarly, miR-21-5p has been validated in the serum of SCI patients, where upregulation correlates with injury severity and inflammatory status. However, its pleiotropic expression across fibrotic disorders and malignancies limits specificity as a standalone SCI biomarker. Oligodendrocyte-enriched miR-219 has also been reported in preliminary human biofluid profiling datasets as a potential surrogate marker of demyelination, although interpretation is complicated by difficulty distinguishing active vesicular secretion from passive leakage after tissue necrosis [89].

In contrast, remaining candidates—including miR-9a-5p, miR-384-5p, miR-124-3p, miR-152-3p, miR-125b-5p, miR-130a-3p, and miR-146a-5p—are largely supported by rodent contusion or ischemia paradigms. Among these, miR-124-3p may have particular translational promise given its CNS enrichment and dual mechanistic relevance as both a neuronal injury marker and an immunomodulator promoting reparative microglial polarization. Nevertheless, its detectability and specificity in human EV cohorts remain to be established, particularly given the broader challenge of confidently assigning cellular origin to circulating EV cargo under systemic inflammation [101,107].

Importantly, this field is characterized by inconsistencies and unresolved negatives. A central limitation is the biofluid–tissue paradox, observed notably with miR-152-3p and miR-384-5p, in which circulating EV levels increase despite local tissue downregulation, complicating interpretation as active signaling versus debris clearance [49,93]. Temporal variability further undermines standardization. For example, miR-21-5p has been reported as upregulated in acute human SCI cohorts, yet other experimental studies demonstrate phase-dependent or context-dependent expression patterns, with divergent functional implications across injury stages [113]. Additional confounders—including age-related inflammaging effects on miR-146a-5p and context-dependent outcomes for candidates such as miR-125b-5p—further caution against direct extrapolation from animal models to human translation [61,104]. Thus, although the diagnostic potential of EV-miRNAs is substantial, clinical implementation will depend on standardized EV isolation protocols and large-scale multicenter validation efforts to resolve expression paradoxes and establish reproducible thresholds (Table 1).

### 4.3. Advanced Technologies for EVs Molecular Profiling

While traditional isolation techniques such as ultracentrifugation and precipitation have served as the foundation for EV research, inherent EV heterogeneity and the complexity of SCI pathophysiology necessitate more sophisticated analytical tools. Emerging technologies have shifted the paradigm from bulk analysis toward high-sensitivity, high-specificity, and single-vesicle profiling. These methodologies offer improved capacity for identifying rare biomarkers, elucidating cargo composition, and distinguishing EV subpopulations derived from specific cellular origins within the CNS (Table 2).

Next-generation sequencing and nucleic acid profiling: Transcriptomic profiling of EVs via next-generation sequencing (NGS) provides a comprehensive view of RNA species carried by EVs, including mRNA, miRNA, lncRNA, and piRNA. This approach is useful for investigating epigenetic and post-transcriptional regulatory mechanisms relevant to SCI. Methodological advances have optimized RNA-sequencing protocols for low-input samples, demonstrating that high-quality EV-associated RNA can be retrieved and sequenced even from archival serum specimens stored for extended periods. Deep sequencing has revealed selective sorting mechanisms whereby specific RNA clusters are preferentially exported into EVs. For example, a large miRNA cluster on chromosome 14q32 has been reported to be exported in EVs while remaining relatively low in parent cells, highlighting distinct EV cargo composition relative to cellular RNA profiles. In addition, sequencing enables identification of expression quantitative trait loci within EVs, suggesting that genetic variation influences EV-associated miRNA content and inter-individual variability in EV cargo [129,130].

RNA-sequencing remains a gold-standard discovery tool rather than a routine clinical diagnostic approach because of cost and turnaround time. However, application to archival samples supports the stability of EV cargo and validates the use of biobanked SCI samples for retrospective biomarker validation. Clinical translation will likely involve using NGS to identify candidate RNA panels and then applying rapid targeted assays (e.g., polymerase chain reaction- or biosensor-based methods) for implementation [129].

Proteomics and multi-omics EV profiling: Mass spectrometry-based proteomics has evolved to address dynamic range challenges inherent to biofluids. While data-dependent acquisition (DDA) has been widely used, data-independent acquisition (DIA) is increasingly used for EV profiling because it fragments ions across defined m/z windows, providing improved reproducibility and coverage of low-abundance proteins. Recent studies have established pipelines that combine efficient isolation methods (e.g., chemical affinity-based EV trap approaches) with DIA phosphoproteomics to quantify thousands of phosphosites. Gas-phase fractionation has been used to generate experiment-specific spectral libraries that can outperform DDA and library-free DIA in identifying and quantifying EV phosphopeptides. In addition, multiplexed DIA incorporating stable isotope labeling (e.g., dimethyl labeling) enables simultaneous analysis of multiple samples, improving throughput and quantitative precision. These proteomic workflows facilitate the identification of dysregulated signaling pathways in pathological states and provide a multidimensional characterization of EV function. Such high-resolution analysis may support differentiation between clinically overlapping entities (e.g., idiopathic and posttraumatic syringomyelia) based on protein signatures related to fibrosis and inflammation [131,132,133,134].

While currently a specialized research tool, DIA phosphoproteomics has demonstrated clinical applicability by stratifying patient risk in cancer cohorts. Its potential for SCI lies in elucidating complex post-injury signaling cascades (e.g., cytoskeletal regulation and inflammation). Transition to routine diagnostics remains challenging because of requirements for high-end instrumentation; however, these workflows serve as a discovery engine for identifying protein targets suitable for simpler immunoassays [131].

Single EV/nanoanalytic technologies: Because bulk analyses average characteristics across heterogeneous vesicle populations, single-vesicle technologies are essential for dissecting EV subpopulations. Single-particle interferometric reflectance imaging sensing, exemplified by the ExoView platform, enables multiplexed characterization of individual EVs. This technology captures EVs on microarray chips using specific antibodies and simultaneously measures size via interferometry and protein profiles via fluorescence, without the need for extensive purification. This approach enables analysis of protein colocalization on single EVs and detection of luminal cargo proteins after permeabilization. Additional advanced imaging techniques include single-particle tracking using total internal reflection fluorescence microscopy (TIRFM) to monitor behavior and subtypes of individual EVs. Super-resolution microscopy techniques, such as direct stochastic optical reconstruction microscopy (dSTORM) and photoactivated localization microscopy (PALM), provide structural resolution of 10–20 nm, enabling visualization of membrane microdomains. In addition, plasmonic scattering microscopy has been developed as a label-free alternative that provides high spatial resolution and signal-to-noise ratios comparable to surface plasmon resonance microscopy, enabling kinetic analysis of antibody binding to single EVs. These nanoanalytic tools are important for validating surface markers and assessing physical heterogeneity of EVs in SCI [38,39,135].

Technologies such as ExoView are commercially available and widely used in translational research, but are not yet standard clinical diagnostic tools. However, single-EV phenotyping is particularly important for CNS injuries in which disease-specific EVs may constitute only a small fraction of the total circulating pool. These technologies therefore have potential as validation tools for confirming biomarker specificity and cell-of-origin signatures in SCI [39].

**Table 2:** Advanced technologies for extracellular vesicle molecular profiling in spinal cord injury research. This table outlines conventional and advanced platforms used for EV molecular profiling in SCI research, highlighting their analytical principles and translational readiness. Abbreviations: EV, extracellular vesicle; SCI, spinal cord injury; DDA, data-dependent acquisition.

	Technique	Mechanism	Advantage	References
1. Next-Generation Sequencing	Deep RNA-sequencing	High-throughput sequencing of small RNAs (miRNA, piRNA) mapping to reference genomes to identify differentially expressed transcripts	Reveals selective export mechanisms (14q32 cluster) and rare transcripts, detects rare transcripts and inter-individual variability better than microarrays	[129,130]
2. Proteomics	Data-independent acquisition (DIA)	Mass spectrometry fragmentation of all ions within defined mass windows	Superior reproducibility and coverage of low-abundance proteins compared to DDA, eliminates stochastic sampling issues	[131,132]
3. Single EVs Analysis	Single particle interferometric reflectance imaging (SP-IRIS)	Captures EVs on antibody-coated chips and images them using interference of reflected light combined with fluorescence	Multiplexed phenotyping and sizing of single vesicles, detects luminal cargo via permeabilization	[38]
	Single-particle tracking (TIRFM)	Real-time tracking of fluorescently labeled individual EVs	Resolves EV heterogeneity and dynamic behavior at the single-vesicle level	[39]
	Super-resolution microscopy (dSTORM, PALM)	Single-molecule localization microscopy using blinking fluorophores to reconstruct images with ~20 nm resolution	Visualizes membrane microdomains and protein clustering on single EVs beyond the diffraction limit of standard microscopy	[39]
	Plasmonic scattering microscopy (PSM)	Uses surface plasmon resonance scattering to image single EVs and quantify binding kinetics label-free	High spatial resolution and signal-to-noise ratio, enables size distribution analysis and protein quantification on individual vesicles	[135]

### 4.4. Limitations of EV-Based Biomarkers in SCI

Despite strong biological rationale and emerging translational promise, several critical limitations currently constrain clinical implementation of EV-based biomarkers for SCI. First, EV heterogeneity and inconsistent nomenclature remain fundamental challenges. Vesicle subtypes—including small and large EVs, microvesicles, and apoptotic bodies—overlap in size, density, and surface markers, while non-vesicular extracellular particles such as lipoproteins and protein–RNA complexes frequently co-isolate with EVs. Without rigorous adherence to MISEV guidelines and orthogonal vesicle characterization, it remains difficult to attribute diagnostic or prognostic signals specifically to vesicular cargo.

Second, pre-analytical and analytical variability substantially limits cross-study comparability. Factors such as blood collection protocols, anticoagulant choice, processing delays, hemolysis, and storage conditions (freeze–thaw cycles, temperature, and duration) can influence EV concentration, size distribution, and RNA/protein cargo stability. These sources of variability may introduce artifactual differences unrelated to SCI pathophysiology, reducing reproducibility across cohorts. In addition, isolation techniques such as ultracentrifugation, precipitation, and filtration differ in yield, purity, and vesicle integrity and may co-isolate lipoproteins or other non-vesicular contaminants. Although advanced technologies—including affinity-based capture, microfluidic platforms, next-generation sequencing, DIA proteomics, and single-EV imaging—offer improved sensitivity and molecular resolution, they remain technically demanding, costly, and largely restricted to specialized laboratories. Standardized, clinically compatible operating procedures are still lacking [136,137,138].

Third, the biological complexity of EV dynamics after SCI introduces important confounders. Circulating EV pools reflect not only CNS-derived vesicles but also substantial contributions from peripheral organs and immune cells activated during systemic inflammatory and acute-phase responses. Consequently, neural EV signals may be diluted or masked, and EV composition varies dynamically over time. Moreover, EV cargo released by neurons, astrocytes, microglia, and oligodendrocytes shifts across injury phases, limiting the interpretability of single-analyte measurements.

Finally, the current biomarker literature is limited by the absence of large-scale, multicenter clinical studies capable of establishing robust correlations between EV-associated cargo and standardized neurological outcomes, such as AIS grade conversion. Accordingly, most reported candidates should presently be regarded as markers of biological plausibility rather than clinically actionable classifiers. Importantly, many of the temporal “windows” described across studies likely reflect limitations in sampling design rather than true peak-expression kinetics and therefore cannot yet be translated into optimized diagnostic decision points. Without comprehensive longitudinal profiling, rigorous adjustment for key clinical confounders (e.g., polytrauma, infection, and surgical intervention), and establishment of reproducible analytical thresholds, it remains premature to determine how reliably specific EV signatures correspond to injury severity or long-term prognosis in real-world clinical practice.

## 5. Conclusions

EVs have emerged as a compelling class of liquid biopsy biomarkers for SCI, offering a distinctive molecular window into the injured CNS. Following SCI, EVs are actively released from neurons, glial cells, endothelial cells, and infiltrating immune cells and can traverse the blood–spinal cord and blood–brain barriers, thereby becoming detectable in accessible biofluids such as plasma, serum, and CSF. Through their protected cargo of proteins and regulatory RNAs, EVs encapsulate dynamic molecular information that reflects tissue damage, neuroinflammation, metabolic stress, and endogenous repair processes. Accumulating evidence suggests that EV-associated structural proteins and inflammatory mediators provide meaningful insight into injury severity, whereas EV-enriched miRNAs encode early molecular signatures linked to neuronal integrity, oligodendroglial dysfunction, secondary injury cascades, and long-term neurological recovery. Collectively, these findings support the premise that EV-based biomarkers may complement conventional imaging modalities and clinical grading scales by enabling earlier diagnosis, more refined stratification of injury severity, and improved prognostication of functional outcomes and secondary complications.

Looking ahead, continued advances in EV isolation methodologies, single-vesicle analytical platforms, and multi-omics integration are expected to facilitate translation of EV-based biomarkers into clinical practice. In particular, integration of advanced nanomaterials—including plasmonic, magnetic, and carbon-based nanostructures—has enhanced analytical sensitivity and specificity. These nanotechnology-enabled platforms permit ultrasensitive detection and high-purity isolation of SCI-specific EV subpopulations, potentially mitigating limitations inherent to traditional approaches in complex biofluids.

Future research should prioritize longitudinal, multicenter validation studies and development of integrated EV protein–miRNA panels to support clinically actionable decision-making in SCI. Importantly, successful translation will require harmonized pre-analytical workflows—including standardized sample handling, hemolysis control, anticoagulant selection, and storage conditions—as well as rigorous EV characterization to ensure cross-study comparability, in accordance with MISEV recommendations. Inter-laboratory ring trials and development of shared reference materials will be essential for establishing reproducibility and defining clinically meaningful cutoff values before EV-based biomarker panels can be implemented at scale [25,139].

## Figures and Tables

**Table 1:** Summary of candidate miRNAs with diagnostic and prognostic potential in spinal cord injury based on serum, CSF, and EV studies. This table summarizes reported EV-associated and circulating miRNAs in SCI, including sample source, study type, diagnostic and prognostic relevance, level of evidence, and major limitations. Evidence tier reflects the strength of available data (clinically validated, supported by preliminary human data, or exploratory preclinical candidate). Abbreviations: SCI, spinal cord injury; EV, extracellular vesicle; CSF, cerebrospinal fluid; AIS, American Spinal Injury Association Impairment Scale; MSC, mesenchymal stem cell.

miRNA	Sample Source	Study Type	Diagnostic Value	Prognostic Value	Evidence Tier	Limitation	Ref
miR-9-3p	CSF EVs	Human (randomized controlled trial cohort), rat	Early detection of CNS injury and astrocyte-derived response after SCI	Predicts spontaneous recovery (AIS grade conversion)	Clinically Validated	Lack of direct evidence for astrocyte-to-neuron transfer in human in vivo models	[97,107,108,109]
miR-21-5p	Serum, spinal neurons, and circulating immune cells	Human (SCI cohort), rat	Extent of tissue destruction and SCI severity (AIS-related) and systemic immune activation	Stage of disease progression and motor functional outcome	Supported by preliminary human data	Low specificity, conflicting expression trends depending on the model	[110,111,112,113]
miR-219	Serum, spinal cord tissue	Human (CSF/Serum); rat	Extent of white matter and myelin injury, SCI severity independent of peripheral trauma	Remyelination potential, long-term neurological recovery, and secondary neurodegeneration/inflammation	Supported by preliminary human data	Difficult to distinguish active vesicular secretion from passive leakage due to oligodendrocyte necrosis	[89,98,99,120,121]
miR-146a-5p	Spinal cord tissue, hUCMSC-derived EVs	Human (MSC-EVs), rat	Loss of endogenous anti-inflammatory feedback in acute SCI	Resolution of inflammation and functional recovery when restored or delivered via EVs	Supported by preliminary human data	Expression is influenced by age, exhibits inverse tissue-biofluid expression patterns	[114,115,116,117,118,119]
miR-124-3p	Spinal cord tissue, MSC-derived EVs, neuron-derived EVs	Mouse, rat	Local gray matter and neuronal damage in injured spinal segments	Functional impairment, recovery, and effectiveness of EV-based neuroprotection	Exploratory preclinical candidate	Needs standardized human serum validation for SCI specifically	[102,122,123]
miR-384-5p	Serum, spinal cord tissue	Mouse, rat	Early grading of SCI severity compared with conventional serum proteins	Depth of secondary injury (ER stress/autophagy) and poorer neurological outcome	Exploratory preclinical candidate	Levels increase in serum but decrease in spinal tissue	[89,90,124,125]
miR-152-3p	Serum EVs, spinal cord tissue, microglia-derived EVs	Mouse, rat	Acute SCI vs. sham and presence of CNS-driven systemic inflammation	Inflammatory burden and risk of SCI-associated cognitive impairments and secondary damage	Exploratory preclinical candidate	Mechanism linking spinal EVs to remote hippocampal dysfunction requires further validation	[49,61,91,93,126]
miR-130a-3p	Serum EVs, lumbar dorsal horn	Rat	Presence of SCI-related neuroinflammation and early indication of NP development	Development and severity of NP and chronic inflammatory injury after SCI	Exploratory preclinical candidate	Primarily studied in rodent pain models	[94,95,96,123]
miR-125b-5p	Serum EVs, spinal cord tissue, microglia-derived EVs	Rat, salamander	Degree of secondary injury (inflammation, neuronal necrosis, failed regeneration)	Functional recovery potential, glial scar formation, and quality of neuroregeneration	Exploratory preclinical candidate	Context-dependent effects	[104,105,106,127,128]
miR-9a-5p	Serum, plasma	Rat	Presence and early severity stratification of acute SCI (AIS A–C)	Burden of primary neuronal damage and potential functional recovery	Exploratory preclinical candidate	Expression trends conflict with human CSF findings	[89,97,101,126]

## Data Availability

No new data were created or analyzed in this study. Data sharing is not applicable to this article.

## Abbreviations

APR	Acute phase response
ASIA	American Spinal Injury Association
AuNPs	Gold nanoparticles
CCR	C-c chemokine receptor type 2
CNS	Central nervous system
CSF	Cerebrospinal fluid
CCL	Chemokine (c-c motif) ligand
DDA	Data-dependent acquisition
DIA	Data-independent acquisition
dSTORM	Direct stochastic optical reconstruction microscopy
ECL	Electrochemiluminescence
ESCRT	Endosomal sorting complex required for transport
EVs	Extracellular vesicles
FRET	Fluorescence resonance energy transfer
GFAP	Glial fibrillary acidic protein
GTPase	Guanosine triphosphatase
HCR	Hybridization chain reaction
HSC	Heat shock cognate
HSPs	Heat shock proteins
ICA	Immunochromatographic assay
IL	Interleukin
ISEV	International Society for Extracellular Vesicles
ILVs	Intraluminal vesicles
LFIA	Lateral flow immunoassays
MRI	Magnetic resonance imaging
mRNA	Messenger RNA
miRNAs	Microribonucleic acid
MVs	Microvesicles
MAPK	Mitogen-activated protein kinase
MVB	Multivesicular body
NP	Neuropathic pain
NGS	Next-generation sequencing
NVEPs	Non-vesicular extracellular particles
NF-κB	Nuclear factor kappa-light-chain-enhancer of activated B cells
OPCs	Oligodendrocyte precursor cells
PALM	Photoactivated localization microscopy
PEF	Plasmon-enhanced fluorescence
pNF-H	Phosphorylated neurofilament heavy subunit
ROS	Reactive oxygen species
S100B	S100 calcium-binding protein B
SEC	Size exclusion chromatography
SPR	Surface plasmon resonance
SCI	Spinal cord injury
SERS	Surface-enhanced Raman scattering
TFF	Tangential flow filtration
TIRFM	Total internal reflection fluorescence microscopy
TBI	Traumatic brain injury
TNF-α	Tumor necrosis factor alpha
UC	Ultracentrifugation
